# Integrating Molecular Dynamics, Molecular Docking, and Machine Learning for Predicting SARS-CoV-2 Papain-like Protease Binders

**DOI:** 10.3390/molecules30142985

**Published:** 2025-07-16

**Authors:** Ann Varghese, Jie Liu, Tucker A. Patterson, Huixiao Hong

**Affiliations:** National Center for Toxicological Research, U.S. Food and Drug Administration, Jefferson, AR 72079, USA; ann.varghese@fda.hhs.gov (A.V.); jie.liu1@fda.hhs.gov (J.L.); tucker.patterson@fda.hhs.gov (T.A.P.)

**Keywords:** SARS-CoV-2, papain-like protease, machine learning, molecular docking, molecular dynamics, drug repurposing

## Abstract

Coronavirus disease 2019 (COVID-19) produced devastating health and economic impacts worldwide. While progress has been made in vaccine development, effective antiviral treatments remain limited, particularly those targeting the papain-like protease (PLpro) of SARS-CoV-2. PLpro plays a key role in viral replication and immune evasion, making it an attractive yet underexplored target for drug repurposing. In this study, we combined machine learning, molecular dynamics, and molecular docking to identify potential PLpro inhibitors in existing drugs. We performed long-timescale molecular dynamics simulations on PLpro–ligand complexes at two known binding sites, followed by structural clustering to capture representative structures. These were used for molecular docking, including a training set of 127 compounds and a library of 1107 FDA-approved drugs. A random forest model, trained on the docking scores of the representative conformations, yielded 76.4% accuracy via leave-one-out cross-validation. Applying the model to the drug library and filtering results based on prediction confidence and the applicability domain, we identified five drugs as promising candidates for repurposing for COVID-19 treatment. Our findings demonstrate the power of integrating computational modeling with machine learning to accelerate drug repurposing against emerging viral targets.

## 1. Introduction

Over the past few years, the world has witnessed a significant surge in emerging and re-emerging infectious diseases, with the coronavirus disease 2019 (COVID-19) pandemic being the most notable example (https://www.cdc.gov/outbreaks/index.html; accessed on 4 February 2025). These types of outbreaks pose a considerable threat to public health and safety, primarily due to their rapid transmission. In such scenarios, the conventional drug discovery process, which spans approximately 10–15 years, is not the most feasible approach to address immediate therapeutic needs. This lengthy timeline highlights the need for alternative strategies, such as drug repurposing [1,2,3], which leverages existing drugs approved by the U.S. Food and Drug Administration (FDA) or other regulatory agencies for new therapeutic applications. Since repurposed drugs have already undergone safety and toxicity evaluations, this approach significantly reduces the time, cost, and risk associated with drug development, making it a viable option for responding to emerging infectious diseases [4].

Recently, drug repurposing approaches using in silico methods have gained widespread popularity due to their ability to significantly accelerate the process by reducing resource requirements [5,6,7,8,9,10,11,12,13,14]. This growing interest has been driven by advancements in technology, the availability of extensive public databases, and progress in medicinal chemistry strategies. Computational methodologies such as artificial intelligence (AI), molecular docking, virtual screening, machine learning, and network pharmacology allow researchers to efficiently screen vast libraries of existing drugs and predict their interactions with novel disease targets [15,16,17,18,19,20,21,22,23]. Notably, integrating these approaches within combined workflows enhances their predictive accuracy and helps overcome the inherent limitations of individual methods, improving the overall success of drug repurposing campaigns [24].

The earliest known example of drug repurposing dates back to the 1980s when aspirin, originally developed as an analgesic, was repurposed as an antiplatelet medication [25]. However, drug repurposing received more attention during the COVID-19 pandemic [26] as researchers worldwide sought rapid solutions to combat this crisis.

The urgency of identifying effective treatments was particularly high given the devastating impact of COVID-19. This necessitated a focus on identifying druggable targets of SARS-CoV-2 to develop effective antiviral therapies. Consequently, the spike (S) protein, RNA-dependent RNA polymerase (RdRp), 3 chymotrypsin-like main protease (3CLpro or Mpro), and papain-like protease (PLpro) were validated as key protein targets for therapeutic intervention against COVID-19 [27,28,29,30].

The S protein, located on the viral surface, facilitates entry into host cells, making it the main target of almost all SARS-CoV-2 vaccines [31,32]. Following infection, RdRp catalyzes viral RNA replication. The structure and mechanism of RdRp are well-characterized, leading to the development of a wide range of RdRp inhibitors [33,34]. Among those deployed against COVID-19 are the repurposed antiviral drugs remdesivir and molnupiravir [35]. Mpro and PLpro are the two cysteine proteases encoded by SARS-CoV-2 responsible for cleaving the polyproteins into individual functional units. Given their critical role in the viral life cycle, both proteases are considered attractive molecular targets for antiviral drug development [36,37,38,39,40,41]. Mpro cleaves the viral polyprotein at eleven conserved sites and has a well-defined catalytic site that serves as the primary target of the FDA-approved oral antiviral drug Paxlovid™. In contrast, PLpro has three proteolytic cleavage sites and, although less structurally characterized compared with Mpro, remains a highly promising target due to its dual roles in viral replication and host immune evasion. It facilitates immune evasion by removing the ubiquitin (Ub) and interferon-stimulated gene 15 (ISG15) post-translational modifications from the host proteins.

Both Mpro and PLpro are cysteine proteases that utilize a catalytic triad composed of cysteine, histidine, and aspartate residues: Cys145–His41–Asp187 in Mpro and Cys111–His272–Asp286 in PLpro. While earlier work described a dyad mechanism, recent structural and computational studies have highlighted the essential role of the aspartate residue in catalysis. This insight is relevant to drug design, as targeting these catalytic residues can enhance inhibitor binding and specificity [42,43].

Despite its diverse functions, PLpro remains underexplored compared with Mpro, which has been widely investigated through in silico methods to identify potential inhibitors [44,45,46,47]. One challenge for discovering selective PLpro inhibitors is due to its similarity to host deubiquitinating enzymes, which raises concerns about off-target effects and toxicity [48]. Numerous high-throughput and virtual screening studies have identified potent PLpro inhibitors, but many of these candidates lack further experimental validation [49,50]. The naphthalene-based compounds like GRL0617 and its derivatives represent a major class of PLpro inhibitors with a well-characterized mechanism of action [43,51].

PLpro inhibitors are primarily accommodated in the substrate-binding sites S3, S4, and SUb2 of SARS-CoV-2 PLpro [52,53,54]. The S1–S4 sites, located near the active site, function as substrate-binding pockets that recognize the ‘LXGG’ tetrapeptide sequence found at the C-terminus of ubiquitin, ISG15, and other viral proteins. The S1 and S2 pockets accommodate the terminal glycines in the LXGG motif and are deeply buried, making them less accessible to inhibitors [53]. As a result, only the S3 and S4 pockets are typically leveraged for inhibitor design. The SUb2 site is a distinct binding pocket located far from the catalytic site and is specifically involved in recognizing ubiquitin (Ub) and ISG15 substrates [52,53].

PLpro contains additional binding pockets, such as SUb1 and the Zn(II) finger region [49,55]. However, these sites remain unexplored in terms of inhibitor development and are not included in this study. More than 50 X-ray crystallographic structures of SARS-CoV-2 PLpro–inhibitor complexes have been reported, and the majority of them have ligands bound in the S3, S4, and SUb2 pockets, highlighting their prominence as the key targets for inhibitor design [52].

While structural and mechanistic insights into PLpro have provided valuable information for drug design [52,56,57,58], research on PLpro is gaining momentum. Recent work by Garnsey et al. demonstrated the successful application of a machine learning-guided pipeline for the discovery of a selective, orally available PLpro inhibitor that showed robust in vivo efficacy in a SARS-CoV-2 mouse infection model [59]. In another study, Pal et al. developed ligand-based machine learning classification models using molecular descriptors and fingerprints to efficiently prioritize potential PLpro inhibitors from large compound libraries [60]. However, no drugs targeting this protease have been approved. As resistance to existing treatments may emerge over time, exploring potential targets such as PLpro remains crucial for long-term therapeutic strategies. Therefore, our study aimed to develop a model by integrating machine learning, molecular docking, and molecular dynamics (MD) simulations for predicting SARS-CoV-2 PLpro binders and non-binders, facilitating the repurposing of FDA-approved drugs for the treatment of COVID-19.

## 2. Results and Discussion

### 2.1. Conformation Dynamics

**Figure 1:**
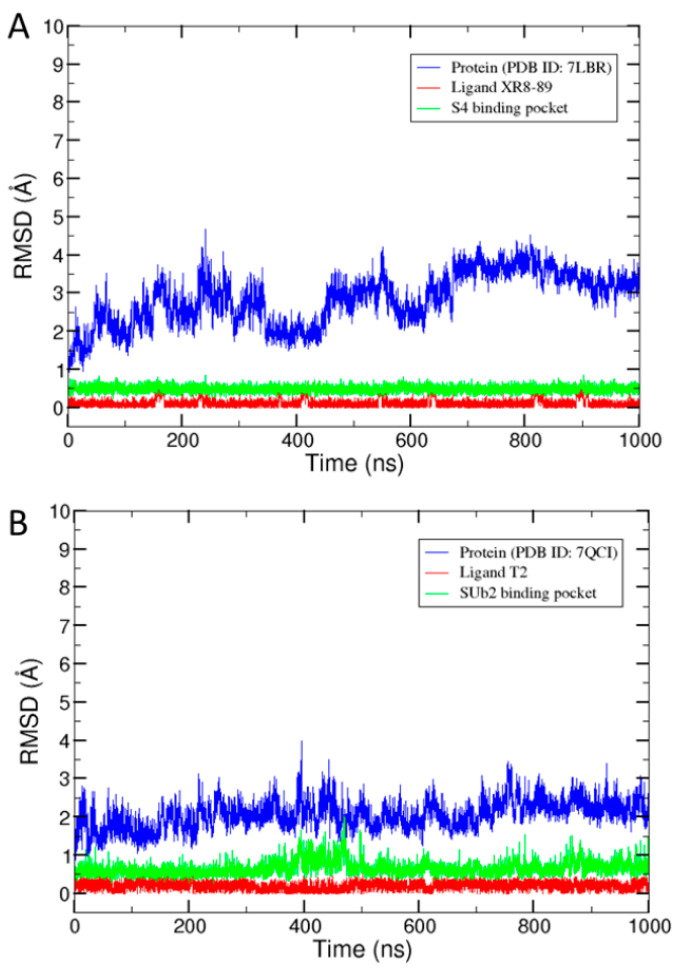
The RMSDs of the structures in the trajectory files from the simulation systems for the PLpro complexes with PDB ID 7LBR (**A**) and 7QCI (**B**). The x-axis indicates the time in the simulation. The y-axis gives the RMSD between the structure at the time indicated on the x-axis and the initial structure. The RMSDs calculated for PLpro, the ligand, and the ligand-binding pocket of PLpro are color-coded in blue, red, and green, respectively.

To examine the stability of the MD simulations, RMSDs were calculated for structures in each of the output trajectory files. The RMSDs over the simulation time for the two simulations are plotted in Figure 1. Our results show that the RMSDs were small throughout the entire simulation process for both systems, indicating that all components of the systems, including PLpro and ligands, were well equilibrated and stable. The PLpro/XT7 complex exhibited an average backbone RMSD of 2.85 ± 0.67 Å, while the PLpro/T2 complex showed a more stable profile, with an average RMSD of 2.06 ± 0.37 Å, suggesting that ligand T2 may confer greater conformational stability to PLpro compared with XT7. Both ligands and their corresponding binding pockets maintained average RMSD values below 1 Å (Figure 1), indicating minimal structural fluctuations within the binding pockets. The RMSDs of the whole PLpro were larger than those of the binding pocket residues [52] and ligands, indicating that the loops and termini of PLpro underwent large conformational changes during the MD simulations due to their structural flexibility. Although the RMSD profiles showed the overall dynamic stability of the two simulation systems, a close examination revealed that molecular conformations during the simulations deviated from the initial structure (RMSD > 0), but many were similar, exhibiting small differences in the RMSD. This observation suggests that PLpro, bound with a ligand, dynamically adopts different conformations in a real biological system. Therefore, assessing the PLpro binding potential for a ligand should not only estimate its binding capability to one specific PLpro conformation, such as that determined in a crystal structure. All distinct groups of PLpro’s dynamic conformations should be used to evaluate its binding potential for ligands.

While this study focused on evaluating ligand-induced conformational changes in PLpro–ligand complexes, we did not include molecular dynamics simulations of the apo (ligand-free) PLpro structure. As a result, we did not perform residue-level root-mean-square fluctuation (RMSF) analysis to compare the binding site flexibility between the apo and bound forms. Although such analysis can provide deeper insights into ligand-induced stabilization or perturbation at the binding site, it falls outside the scope of this work, which primarily focused on the high-throughput identification of potential PLpro binders through an integrated docking and machine learning framework. Future studies incorporating apo simulations and RMSF comparisons would be valuable for further elucidating the dynamic behavior of PLpro in the absence and presence of candidate inhibitors.

### 2.2. Representative Structures

**Figure 2:**
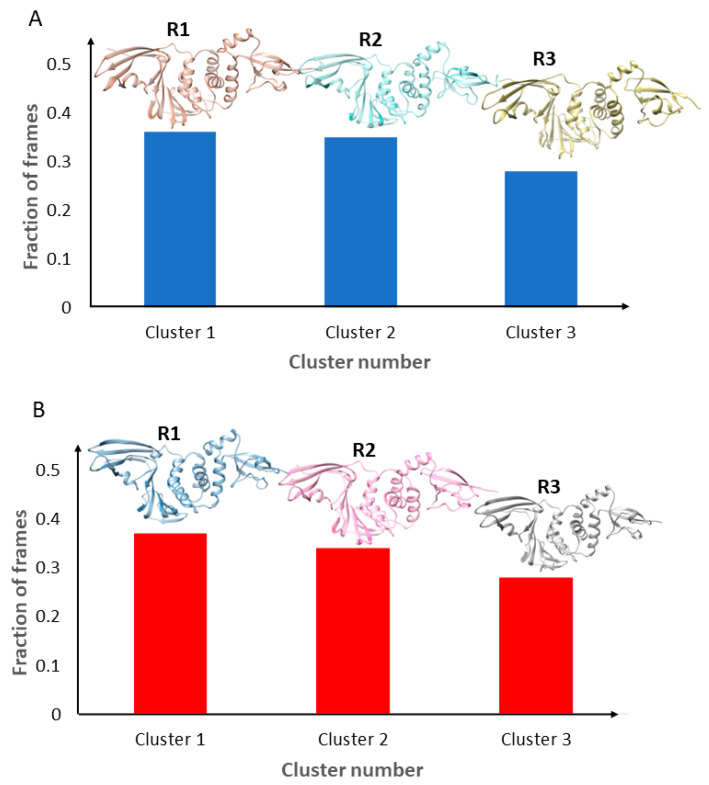
The structure clusters and corresponding representative structures from the MD simulations based on the PLpro complexes with PDB ID 7LBR (**A**) and 7QCI (**B**). The y-axis of the bar graph denotes the fraction of structures, and the x-axis indicates the cluster number. The representative structures, marked as R1, R2, and R3, are shown on top of the corresponding bars.

To identify representative structures for enhancing the evaluation of the PLpro binding of compounds, the unsupervised machine learning algorithm K-means clustering was used to group structures in the trajectory files into three clusters based on their structural similarity. Figure 2 gives the numbers of structures in these clusters and the corresponding representative structures. For the simulation using PLpro with PDB ID 7LBR, clusters 1, 2, and 3 contained 36.3%, 35.0%, and 28.6% of the structures in the output trajectory, respectively, as shown in Figure 2A. The relatively balanced cluster distribution suggests that the system explored multiple conformations without the dominance of one state. The average RMSD compared with the representative structure for all structures in each cluster was 2.06 Å (±0.44 Å standard deviation), 1.80 Å (±0.39 Å), and 2.19 Å (±0.25 Å), indicating the structures in each cluster were similar to the corresponding representative structure.

A similar clustering pattern was observed for the simulation based on the structure with PDB ID 7QCI, where clusters 1, 2, and 3 accounted for 37.6%, 34.2%, and 28.2% of the output structures in the trajectory file, respectively, as depicted in Figure 2B. The mean RMSD values of the structures based on their respective representative structures were 1.55 Å, 1.57 Å, and 1.49 Å, with associated standard deviations of 0.26 Å, 0.27 Å, and 0.22 Å, respectively, again indicating the structures in each cluster were similar to their representative structures.

For both simulations, the representative structures implied the key conformational states explored during the MD simulations. These representative structures play important roles in comprehensively estimating the PLpro binding potential of compounds; therefore, they were used in our molecular docking study.

### 2.3. Molecular Docking

The six representative structures generated from the clustering analysis of the MD simulation trajectory files were used to assess the PLpro binding potential of compounds through molecular docking. The PLpro structures were set to be rigid, while the ligands were flexible to explore different conformations during docking. For each ligand, the top five docking scores were output for each of the six representative structures, resulting in thirty docking scores, which corresponded to thirty docking poses.

**Figure 3:**
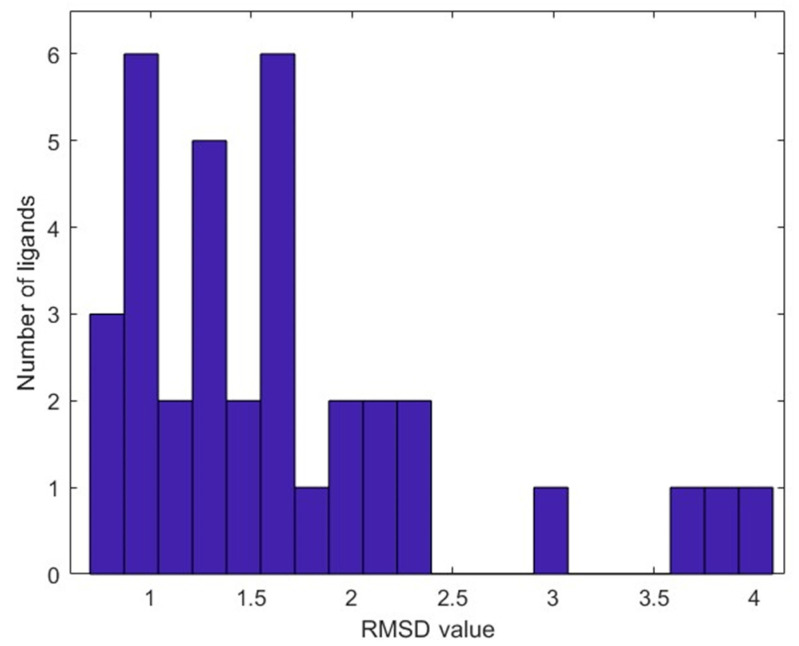
The distribution of RMSD values between the docked poses and the corresponding co-crystallized conformations of the 33 ligands obtained from PDB.

To assess the reliability of the molecular docking protocol, we evaluated its ability to reproduce experimentally observed ligand binding conformations. The resulting docked complexes were superimposed with the original co-crystallized structures based on the backbone atoms of PLpro, and the RMSD values between the docked and experimental ligand poses were calculated and are provided in Supplementary Table S1. As shown in Figure 3, most of the ligands exhibited close agreement with their co-crystallized conformations, with an average RMSD of 1.68 Å. This result confirmed that the docking protocol reliably recapitulated the experimentally observed binding modes and was suitable for use in our study.

**Figure 4:**
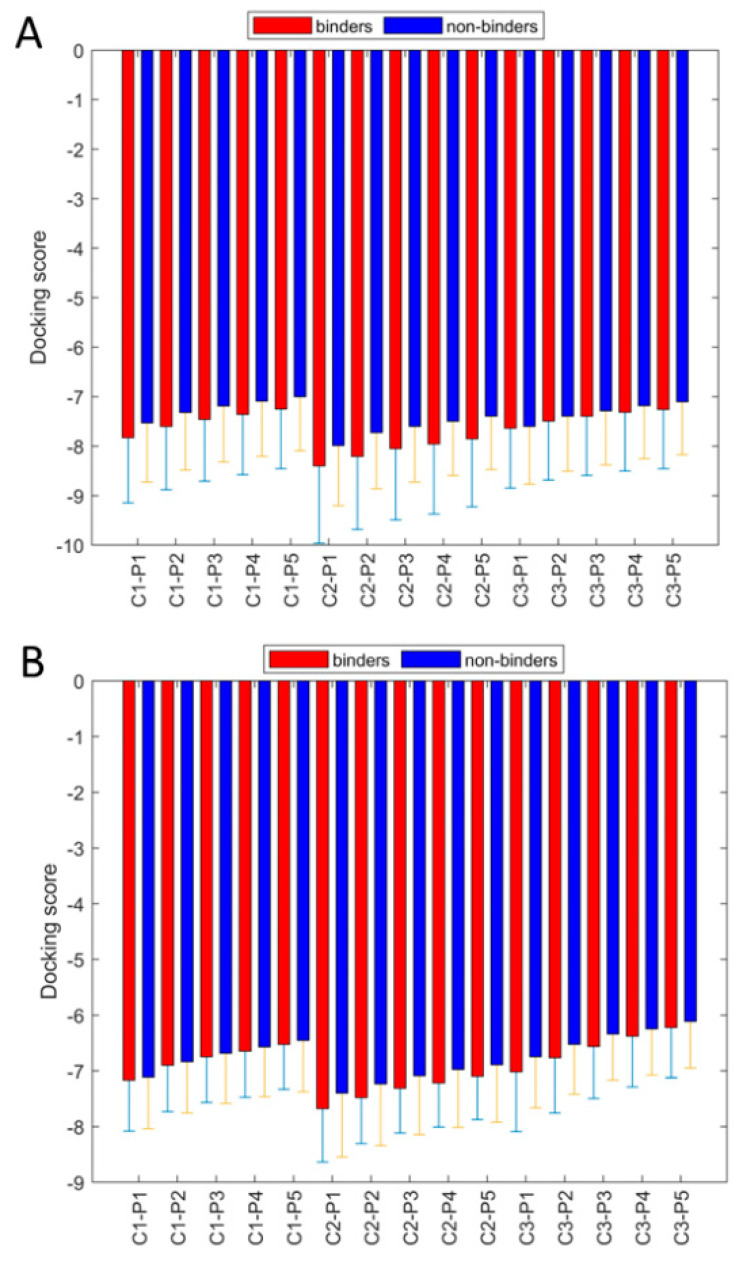
The docking scores of 127 training compounds from the docking to site S4 (**A**) and site SUb2 (**B**). The average docking scores of 58 binders and 69 non-binders are plotted as red and blue bars, respectively, for the top five docking poses for each of the six representative structures labelled on the x-axes. The corresponding standard deviations are depicted by the attached sticks. The x-axis labels are given in a combination of representative conformations (C1 to C3) and top poses (P1 to P5).

The resulting docking scores of the 127 training compounds and the 1107 FDA-approved drugs are provided in Supplementary Tables S2 and S3, respectively. Figure 4 summarizes the docking scores obtained from the 127 training compounds. The binders statistically exhibited lower docking scores compared with the non-binders in docking at both S3 and S4 (termed as binding site S4 hereafter) and SUb2 sites, indicating that molecular docking analysis could be used to differentiate PLpro binders and non-binders. However, as shown in Figure 4, the standard deviations were larger than the differences between the mean docking scores for the corresponding binders and non-binders, suggesting that using a single representative PLpro structure with a clear cut-off docking score to differentiate binders from non-binders was not feasible. Thus, a more sophisticated method, such as machine learning, was needed to pinpoint the complex pattern in the docking scores from all representative structures and multiple docking scores for predicting the PLpro binding potential of compounds.

To evaluate the usefulness of the docking scores from our docking procedures in PLpro binding prediction, the docking scores of the 33 ligands derived from PLpro complexes with binding in the S4 and SUb2 sites, as reported in the PBD database, were used to predict their likely binding sites. The average of 15 scores from docking a ligand to the S4 site of the three representative structures was compared with the average value from docking to the SUb2 site to predict the likely binding site of the ligand. The results are shown in Table 1. The binding sites of 30 of the 33 ligands were correctly predicted, leading to an overall prediction accuracy of 91%. As marked in bold in Table 1, two ligands (9EI and L30), which bind at the S4 site in the structures from the PDB database, had a preference for docking at the SUb2 site to the S4 site and, thus, were incorrectly predicted to bind at SUb2 by our molecular docking. Meanwhile, A3X, which experimentally showed binding at the SUb2 site, was predicted to bind at the S4 site by docking analysis. The results demonstrate that our molecular docking analysis is useful to reliably predict PLpro binding for compounds.

**Figure 5:**
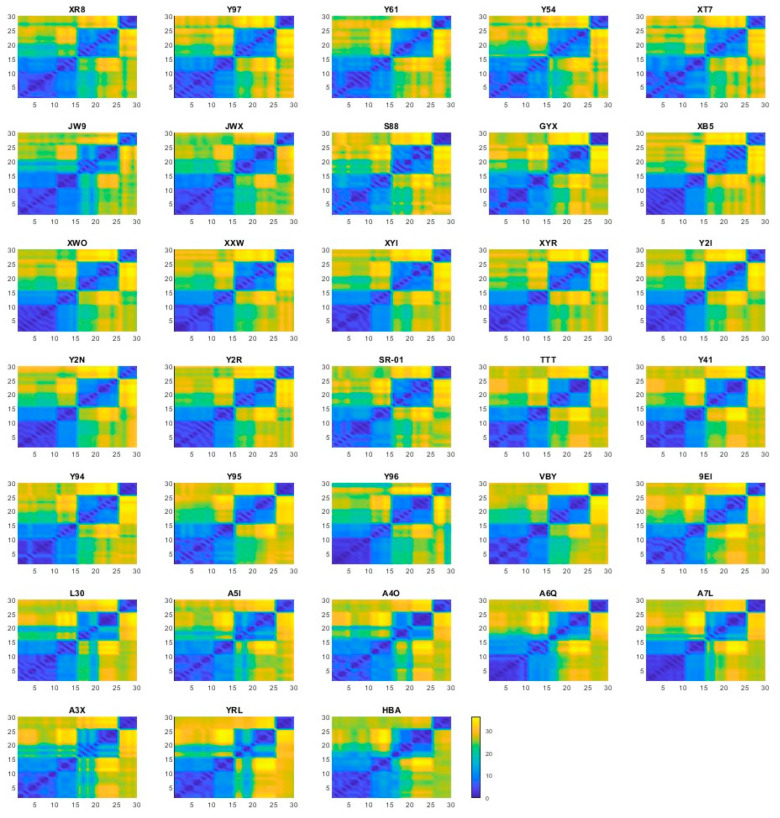
The matrices of the RMSD values between the 30 docking poses for each of the 33 ligands. Each subfigure corresponds to one ligand, with the ligand name displayed above. The x-axis and y-axis represent the pose indices. The first 15 poses correspond to the S4 binding site and the remaining 15 to the SUb2 site. Within each site, the poses are grouped by the representative protein conformation used for docking: five poses from the first representative structure, followed by five from the second, and five from the third.

Since each ligand was docked into the S4 site of three representative PLpro structures and into the SUb2 site of three additional representative structures—yielding five poses per docking—we evaluated the structural similarity among the resulting 30 poses for each ligand using root-mean-square deviation (RMSD). The RMSD similarity matrices for all 33 ligands are shown in Figure 5, with detailed values provided in Supplementary Table S4. As observed, the docking poses generated from the same representative structure exhibited high structural similarity, reflected by the low RMSD values. In contrast, as expected, poses from the S4 and SUb2 binding sites showed marked differences due to the distinct nature of the two sites. These results support the use of averaged docking scores across multiple conformations as a reasonable approach to account for structural variability in binding site interactions.

Although docking scores provide a useful approximation of binding affinity, they are derived from simplified energy functions that may not fully account for the dynamic and complex nature of protein–ligand interactions. To reduce the risk of overinterpreting individual docking outcomes, the top five scoring poses were used for each ligand across multiple protein conformations. This strategy helped to capture the variability in ligand binding and provided a more robust basis for downstream analysis.

### 2.4. Model Performance

**Figure 6:**
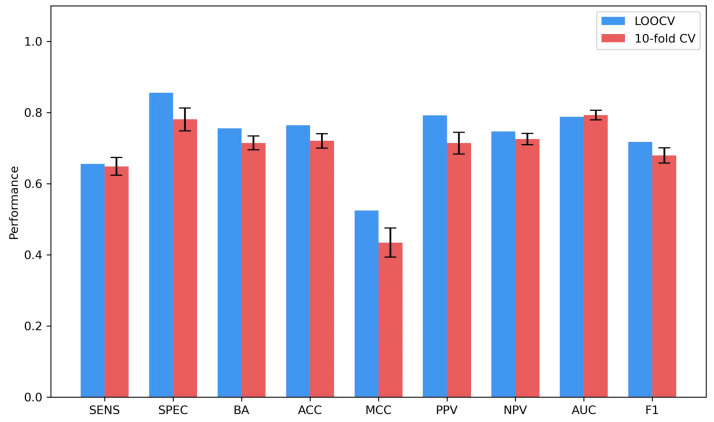
The results of LOOCV and 10-fold cross-validations. The x-axis indicates the performance metrics, and the y-axis depicts the metric values. The LOOCV results are presented as the blue bars. The average performance metrics values from the 100 iterations of 10-fold cross-validations are given as the red bars, and their corresponding standard deviations are indicated by the sticks atop. Abbreviations: Sens—sensitivity, Spec—specificity, BA—balanced accuracy, Acc—accuracy, MCC—Matthews’ correlation coefficient, PPV—positive predictive value, NPV—negative predictive value, AUC—area under the receiver operating characteristic curve, and F1—F1 score.

To evaluate the performance of the model trained using the 30 docking scores for predicting the PLpro binding potential of compounds, both LOOCV and 100 iterations of 10-fold cross-validation were conducted on the 127 training compounds. Figure 6 illustrates the LOOCV and 10-fold cross-validation results. The LOOCV-based models achieved good overall performance, with balanced accuracy, accuracy, MCC, area under the receiver operating characteristic curve (AUC), and F1 scores of 0.755, 0.764, 0.524, 0.788, and 0.717, respectively. In comparison, the average performance metrics from the 10-fold cross-validation were slightly lower—0.715 (balanced accuracy), 0.720 (accuracy), 0.434 (MCC), 0.793 (AUC), and 0.679 (F1-score)—but the differences were not statistically significant (*p* = 0.3786; paired t-test). Additionally, the standard deviations across the 100 iterations were small, indicating that the RF models are stable across different data partitions. Based on these findings, we retained LOOCV for reporting model performance in subsequent analyses.

Interestingly, the specificity (0.855) was higher than the sensitivity (0.655), indicating the models had better performance in predicting non-binders than in predicting binders. This may be attributed to the higher preference of non-binders (69) compared with binders (58) in the training dataset.

**Figure 7:**
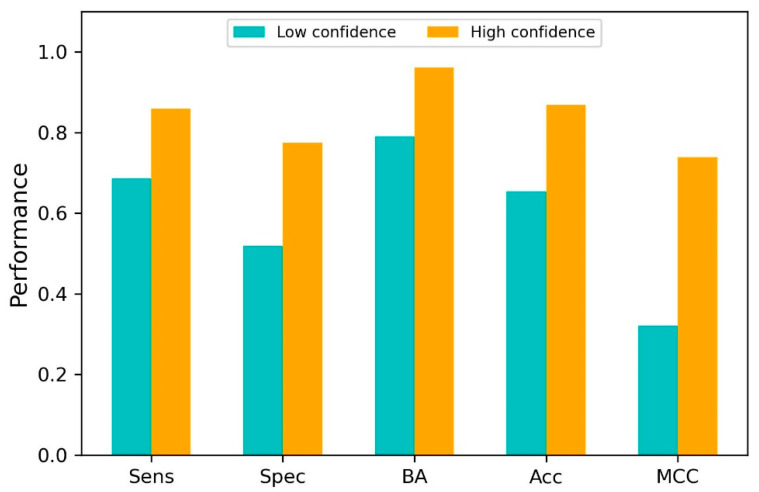
The relationship between prediction confidence and performance in LOOCV. The low-confidence and high-confidence predictions are plotted in the cyan and yellow bars, respectively. The x-axis shows the performance metrics, and the y-axis gives the metric values. Abbreviations: Sens—sensitivity, Spec—specificity, BA—balanced accuracy, Acc—accuracy, and MCC—Matthews’ correlation coefficient.

The RF model not only predicted if a compound was a PLpro binder or non-binder but also gave a probability to quantify the likelihood of the compound being a binder or non-binder. This probability value could be used to measure the confidence of the prediction. To assess the usefulness of the confidence measurement in applying the RF model, confidence values were calculated, and their relationship with prediction performance was examined. As shown in Figure 7, high-confidence predictions outperformed low-confidence predictions. The results indicate that prediction confidence provides useful information for the appropriate use of predictions from the RF model.

The member tree models in an RF model use a subset of the independent variables, which were the 30 docking scores in this study. RF provides information on the importance of individual docking scores to the constructed model. To identify docking scores important for predicting the PLpro binding potential, the importance values for each docking score from the 127 RF models in the LOOCV were added up, resulting in a total importance value. Statistically, the total importance of each docking score would contribute 3.3% if the 30 docking scores were equally important to the RF models. There were 11 docking scores that exhibited more than the average 3.3% importance and accounted for 70% of the contributions, as listed in Table 2, indicating that they were statistically important to the RF models. Interestingly, of the 11 docking scores, 8 were the results from docking to the S4 sites, demonstrating the preference of ligands at this site, which is consistent with the number of experimentally determined structures in the PDB database. Furthermore, representative structures from different clusters and all top five docking scores were included in this list, suggesting dynamic structural clusters and multiple top docking scores should be considered to build a machine learning model for predicting PLpro binding as implemented in this study.

The feature importance analysis revealed that docking scores associated with the S4 site across several representative protein conformations contributed the most to model performance. This implies that compounds forming strong interactions with the S4 site are more likely to exhibit PLpro inhibitory activity. These insights can inform the prioritization of candidate compounds during virtual screening by focusing on molecules that score well against the most predictive structures and binding sites identified by the model. In this way, the model not only offers predictive capability but also provides interpretable guidance for the rational design or selection of new chemical entities targeting PLpro.

The applicability domains of the predictions in the LOOCV were calculated and analyzed to examine their relationship with prediction performance. The Euclidean distances of the predicted compounds to the centroid of the training set in the space represented by the 30 docking scores were first calculated. The prediction accuracy for 95 predictions (75%) that were near the centroid (with a distance shorter than 4.713 kcal/mol) was 77.9%, higher than the prediction accuracy for the other 32 predictions that had distances to the centroid longer than 4.713 kcal/mol. This finding indicates that the applicability domain measured by the Euclidean distance to the centroid of the training compounds offers useful information for assessing the reliability of predictions from the constructed RF model.

One limitation of this study was the absence of an independent external dataset for model validation. While LOOCV and 10-fold cross-validations were employed to make the most efficient use of this small dataset, external testing is crucial for evaluating model generalizability. Unfortunately, to our knowledge, no publicly available dataset with experimentally validated PLpro binders and non-binders currently exists. As such, external validation was not feasible within the scope of this work. Future studies incorporating independent datasets, as they become available, will be important to further assess and refine the model’s predictive performance in broader applications.

### 2.5. Identification of Drugs as Candidates for Repurposing

**Figure 8:**
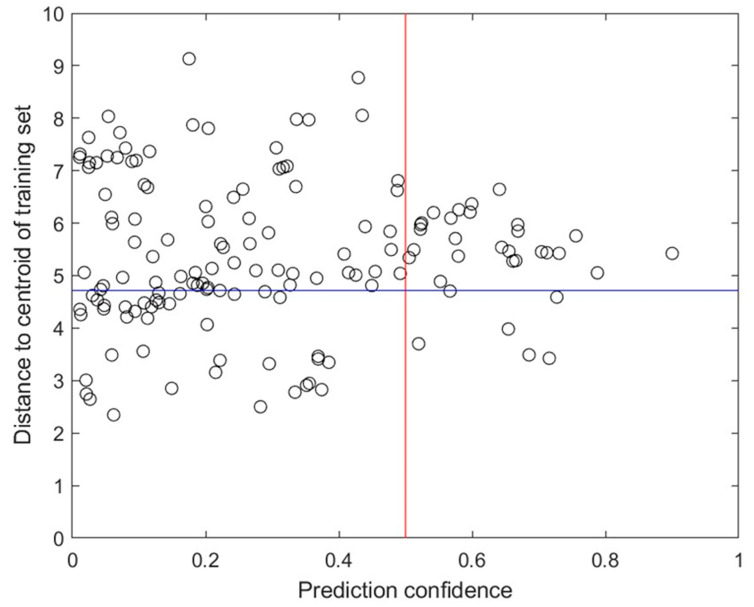
FDA-approved drugs predicted as PLpro binders. The x-axis represents the prediction confidence value. The y-axis shows the distance to the centroid of the training dataset. The drugs are plotted as circles. The vertical line indicates the prediction confidence value of 0.5. Points to the right of the vertical line represent high-confidence predictions, while those to the left indicate low-confidence predictions. The horizontal line separates the drugs inside and outside the applicability domain: points below the line fall within the applicability domain, whereas points above the line are outside the domain.

To identify candidates to repurpose for COVID-19 treatment through targeting PLpro, the RF model constructed from the 30 docking scores of the 127 training compounds was applied to predict the 1107 FDA-approved drugs for their PLpro binding potential using their docking scores. Of the 1107 drugs, 146 were predicted to be PLpro binders. As shown in Figure 8, the predictions of six drugs showed high prediction confidence (≥0.5) and were inside the applicability domain (with a distance to the centroid of the training compounds of <4.713 kcal/mol), indicating that they were very likely PLpro binders. Among these six drugs, troglitazone, marketed as Rezulin, has been withdrawn from the market due to hepatotoxicity. The other five drugs, listed in Table 3, are drugs on the market and may serve as promising candidates for drug repurposing for COVID-19 treatment.

The drugs to date that have been approved by the FDA to treat COVID-19 target different proteins of SARS-CoV-2. Nirmatrelvir, the main component of Paxlovid, which was approved in May 2023, targets SARS-CoV-2’s main protease. Remdesivir and molnupiravir were approved by the FDA in October 2020 and December 2021, and both inhibit SARS-CoV-2 RNA-dependent RNA polymerase. Emergency use authorization was granted to baricitinib in November 2020, but it does not target SARS-CoV-2. The five drugs identified in this study as potential drug repurposing candidates for COVID-19 treatment likely target PLpro, providing potential new treatments complementary to current treatment options if they are successful in clinical validation.

Notably, several of the identified drugs have been reported as potential binders of SARS-CoV-2 Mpro in prior in silico studies. Comoglicic acid has also been experimentally validated as an Mpro inhibitor. These findings suggest that the identified drugs possess broader antiviral activity, which supports their repurposing potential and warrants further experimental investigation of their efficacy against PLpro.

The random forest model developed in this study demonstrated high specificity (0.855), indicating a strong ability to correctly identify non-binders, but only moderate sensitivity (0.655), which may result in a higher rate of false negatives. This trade-off suggests that while the model is reliable in ruling out unlikely candidates, it may miss some true binders. To improve hit recovery, future work could explore tuning the decision threshold to prioritize sensitivity or applying probabilistic calibration techniques to better balance sensitivity and specificity. Such adjustments could help minimize false negatives, which is particularly important in early-stage virtual screening, where identifying as many potential hits as possible is desirable for downstream experimental validation. These enhancements may further improve the model’s utility in drug repurposing efforts targeting PLpro.

## 3. Materials and Methods

### 3.1. Study Design

**Figure 9:**
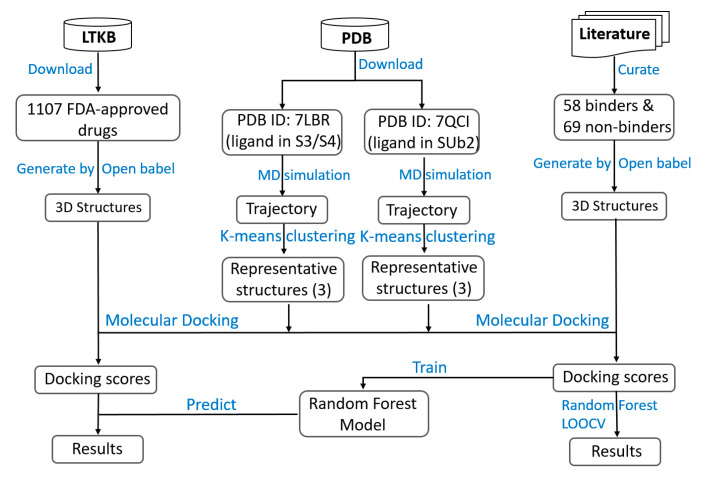
Study design. Two PLpro structures were downloaded from the Protein Data Bank (PDB) and subjected to molecular dynamics (MD) simulations. The resulting trajectories were clustered using k-means clustering to generate representative structures. Ligands classified as binders or non-binders were curated from the PDB and the literature, while the structures of FDA-approved drugs were sourced from the LTKB database. All compounds were docked into the representative PLpro structures using Autodock Vina. The docking scores of all representative structures were used to develop a random forest (RF) classification model. The performance of the model was then evaluated using leave-one-out cross-validation (LOOCV). The final RF mode was then used to predict potential PLpro binders from FDA-approved drugs, identifying candidates for possible COVID-19 treatment via drug repurposing.

The study design is illustrated in Figure 9. Two three-dimensional (3D) structures of PLpro were retrieved from the protein databank (PDB): 7LBR [61] and 7QCI [55]. The structures were then subjected to MD simulations to identify representative conformations for subsequent molecular docking studies. The structures in the resulting MD simulation trajectories were clustered using the K-means algorithm, yielding three clusters for each trajectory. A representative structure was generated from each cluster and was used for molecular docking.

A set of 127 ligands was curated from the literature, consisting of 58 binders (including 33 ligands from crystallographic structures in the PDB) and 69 non-binders. The 3D structures of the ligands were generated using Open Babel-v3.1.1 [62] and then docked in the six representative PLpro structures using Autodock Vina-v1.2.3 [63]. The docking scores from the top five docking poses were output for docking a compound to each of the six representative structures. This resulted in 30 docking scores per compound, which were used as input features for subsequent machine learning model development. A random forest (RF) model was trained on the docking results of the 127 ligands. The model was validated using leave-one-out cross-validation (LOOCV) to assess its predictive performance and then applied to 1107 FDA-approved drugs curated from the LTKB (Liver Toxicity Knowledge Base) [64] to identify potential SARS-CoV-2 PLpro binders.

### 3.2. MD Simulation System Preparation

The 3D structures of SARS-CoV-2 PLpro complexed with 5-[(azetidin-3-yl)amino]-N-[(1R)-1-{3-[5-({[(1S,3R)-3-hydroxycyclopentyl]amino}methyl)thiophen-2-yl]phenyl}ethyl]-2-methylbenzamide (XT7) in the S4 substrate-binding site (PDB ID: 7LBR; chain B; 2.20 Å) and N-(3,4-dihydroxybenzylidene)-thiosemicarbazone (T2) in the SUb2 substrate-binding site (PDB ID: 7QCI; chain A; 1.76 Å) were downloaded from the PDB website (RCSB PDB: Homepage). These structures were selected based on criteria resolution, amino acid mutations, and the availability of publications detailing crystallization conditions. The first criterion ensured structure quality. The second criterion warranted that the selected structures were close to their native forms and did not have any alterations that could affect ligand binding or conformational dynamics. Though the selected structures did not have mutations, 7QCI had a modified cysteine residue (Cys111), which was changed to its original form before performing MD simulations. The third criterion ensured enough technical information for understanding the structures.

The missing atoms, including hydrogens, in the downloaded structures were added using the Leap module in AMBER 16 [65]. The structures were optimized using the Amber ff14SB force field [66]. The systems for MD simulations were generated by adding counterions for neutralization and were solvated with the standard TIP3P water model [67] in a rectangular box with a 10 Å buffer around the proteins. The tetracoordinated Zn(II) ion in the finger region was described by the zinc amber force field (ZAFF) [68]. The missing hydrogen atoms in the ligands XT7 and T2 were added using Chimera-v1.18 [69]. AMBER atom types were used for the ligands. The partial atomic charges were generated for the ligands using the AM1-BCC charge method [70] in Antechamber.

### 3.3. MD Simulations

After preparation, the systems underwent two-step energy minimization to alleviate steric clashes, which stabilized the systems. In the first step, only solvent molecules were minimized, and proteins remained unchanged. During step two, both solvent and protein molecules were minimized. The minimization was conducted using 5000 cycles with the steepest descent algorithm, followed by another 5000 cycles with the conjugate gradient method. After minimization, the systems were gradually heated from 0 K to 300 K for 100 ps using a Langevin thermostat [71]. Following heating, the systems were equilibrated. Density equilibration was first performed for 1 ns under constant temperature and pressure to allow the systems to achieve density stabilization (1 gm/cm^3^). The pressure was controlled by a Berendson barostat [72]. A 3 ns equilibration was then conducted to further relax the systems at the stabilized density. Subsequently, a production run of 1 µs was performed for each system with a timestep of 2 fs under periodic boundary conditions. All covalent bonds involving hydrogens were constrained using the SHAKE algorithm [73]. The MD simulations were carried out in the Particle Mesh Ewald Molecular Dynamics (PMEMD) module in AMBER. The stability of the resulting trajectories was analyzed by calculating the root-mean-square deviation (RMSD) in the CPPTRAJ module [74] in AMBER.

Each trajectory consisted of 10,000 frames. The structures of PLpro in each trajectory were clustered into groups based on the RMSD values of Cα atoms using the K-means clustering algorithm available in CPPTRAJ [74]. The number of clusters was set to three. A representative PLpro structure was determined from each cluster. The RMSD values between the PLpro structures in a cluster were calculated first. The structure with the lowest average RMSD values to other structures in the same cluster was deemed the representative structure for the cluster and was used in subsequent molecular docking. The PDB files of the six representative structures of PLpro are provided in the Supplementary Data.

### 3.4. Prepare Protein and Ligand Structures for Molecular Docking

The PDB files of representative PLpro structures from MD simulations were converted to Autodock-compatible input file (PDBQT) format using Autodock Tools-v1.5.7. Three groups of compounds were used in the molecular docking: 33 ligands contained in the structures from the PDB, 94 compounds whose binding activity to PLpro had been experimentally tested (25 binders and 69 non-binders), and 1107 FDA-approved drugs. Non-binders were defined as compounds for which IC_50_ values were either not determined or reported as ‘none’, typically due to a lack of measurable inhibition in primary screening assays. Binders were identified from co-crystallized ligands bound in PDB and curated from literature sources where IC_50_ values were experimentally determined.

The 3D structures of the 33 ligands (Table 4) from the PDB were obtained by directly separating them from the downloaded complex structures. The two-dimensional (2D) structures of the 94 compounds with experimental data and the 1107 drugs were first downloaded from PubChem or drawn using ChemDraw-v20.1 when not available in PubChem, and were then converted to 3D structures using Open Babel [62]. All structures were protonated based on physiological pH and subjected to energy minimization using the default parameters in Open Babel (MMFF94 forcefield; 100-step steepest descent; 10-step conjugate gradient). The optimized structures were output in MOL2 format by Open Babel and subsequently converted to PDBQT format using Autodock Tools-v1.5.7. The PDBQT files of the training compounds and the 1107 drugs are provided in the Supplementary Data.

### 3.5. Molecular Docking

The molecular docking of the compounds into the representative structures of PLpro was carried out using AutoDock Vina [63]. A docking box was first defined for each of the representative structures, with its center at the centroid of the reference ligand (XT7 or T2). To determine the docking box dimension for the docking compounds into substrate-recognizing sites S4, residues within 3.5 Å of the 26 ligands in the structures of the PLpro complexes obtained from the PDB (Table 4) were considered as interacting residues. In a similar way, interacting residues were determined using the 7 ligands bound at the SUb2 site for defining the docking box to dock compounds to the SUb2 site. The docking boxes were then set to enclose the interacting residues in the six representative structures. The dimensions of the docking boxes are provided in Table 5.

Molecular docking was performed using an exhaustive search setting of 32 and an energy range of 3. During the docking process, the structure of PLpro was kept rigid while ligand conformations were explored. The five lowest docking scores, along with their corresponding conformations, were output for each docking analysis.

### 3.6. RF Model Development

Though many machine learning algorithms, such as support vector machine and decision forest [79,80], are available, we selected RF due to its robustness against overfitting by averaging multiple decision trees [81,82]. RF is an ensemble learning algorithm that combines predictions from multiple decision trees constructed on subsets of samples and independent variables randomly selected from an entire training dataset [82]. An RF model was developed based on the molecular docking results of the 58 binders and 69 non-binders for identifying PLpro binders from the 1107 FDA-approved drugs as potential candidates for repurposing to treat COVID-19. The performance of the model was evaluated using leave-one-out cross-validation (LOOCV).

During the construction of the RF models, key algorithmic hyperparameters, including *n_estimators*, *min_samples_split*, and *min_samples_leaf*, were tuned, while default values were used for the rest of the hyperparameters. Multiple tree numbers (50, 100, and 200) and various combinations of minimum node size and minimum split size (1 and 2, 2 and 4, 3 and 6, 4 and 8, 5 and 10, and 6 and 12) were explored. The best configuration, determined by the model performance, consisted of 200 trees, a minimum node size of 1, and a minimum split size of 2. These tuned hyperparameters were then used to train the final RF models.

The development, validation, and prediction of the RF models were conducted using the packages in Scikit-learn (0.23.2) [83] in Python (https://www.python.org/downloads/release/python-385/ (accessed on 16 May 2024)).

### 3.7. Model Performance Measurement

The performance of the RF models was measured using accuracy, sensitivity, specificity, balanced accuracy (BA), and Matthews’ correlation coefficient (MCC), which were calculated using Equations (1)–(5):

(1) $$Accuracy=\frac{TP+TN}{TP+TN+FP+FN}$$

(2) $$Sensitivity=\frac{TP}{TP+FN}$$

(3) $$Specificity=\frac{TN}{TN+FP}$$

(4) $$BA=\frac{Sensitivity+Specificity}{2}$$

(5) $$MCC=\frac{TP*TN-FP*FN}{\sqrt{\left(TP+FP\right)*\left(TP+FN\right)*\left(TN+FP\right)*\left(TN+FN\right)}}$$

where TP represents the number of true positives, TN means the number of true negatives, FP denotes the number of false positives, and FN indicates the number of false negatives.

### 3.8. Prediction Confidence

The RF models not only predicted a compound as a PLpro binder or non-binder but also output a prediction probability to quantify the likelihood of the compound being a binder, which indicated the confidence of the prediction. Prediction confidence analysis has proved to be a valuable metric for the application of various machine learning models [84,85,86]. In this study, prediction confidence was calculated using Equation (6):

(6) $$Prediction\;confidence=\frac{\left|prob-0.5\right|}{0.5}$$

where prob is the probability of a compound predicted as a PLpro binder.

The relationship between predictions in the LOOCV and their prediction confidences was analyzed. Predictions were first divided into two groups: low-confidence predictions, with a prediction confidence of <0.5, and high-confidence predictions, with a prediction confidence of ≥0.5. The performance metrics were then calculated for the predictions in each group.

### 3.9. Identification of Important Docking Scores

The 30 docking scores used in model development were obtained from the molecular docking of compounds into the six representative PLpro structures. Each score contributed differently to predicting a compound’s likelihood of being a PLpro binder and may have, therefore, played a distinct role in the performance of the RF models. To identify the important docking scores for the RF models, the use frequency of the 30 docking scores in the RF models during LOOCV was analyzed. The frequently used docking scores were considered informative to the RF models and were important for PLpro binding. We first added up the importance values from all the models in the LOOCV for each docking score. The sums of the importance values were then used to identify the docking scores important for training the RF models. The docking scores with sums of importance values larger than the statistical average were deemed to be important for training the models.

### 3.10. Applicability Domain Analysis

The applicability domain defines the boundaries of chemical structures used to train a model and is an important metric for appropriately utilizing the model [87,88]. Various methods can be used to define a model’s applicability domain. In this study, we used the centroid of training compounds in the space represented by their docking scores as a reference point for measuring the applicability domain of the trained model. Compounds located nearer to the centroid are more likely to reside within the applicability domain of the trained model. We used the distance that covered 75% of the 127 training ligands as the cut-off for determining if a compound was inside or outside the applicability domain when applying the training model to predict the 1107 FDA-approved drugs for identifying highly potential PLpro binders for repurposing to treat COVID-19.

The scripts and code used for building the machine learning models, performing molecular docking, and conducting MD simulations are provided in the Supplementary Information.

## 4. Conclusions

In summary, this study demonstrated the power of combining molecular simulations, docking, and machine learning to identify potential drug candidates targeting SARS-CoV-2 PLpro, an underexplored but vital target. By simulating the dynamics of PLpro structures with ligands bound at two distinct sites and leveraging molecular docking to build a PLpro binding activity prediction model using the machine learning algorithm random forest, we successfully identified five FDA-approved drugs to be PLpro binders with high confidence and inside the model’s applicability domain, providing candidates to repurpose for COVID-19 treatment. This approach of integrating machine learning and computational chemistry showcases a powerful framework for the identification of current drugs for repurposing to treat other diseases for future drug discovery efforts. As PLpro has dual roles in viral replication and immune suppression, targeting this enzyme could yield therapeutic benefits beyond viral load reduction. The findings suggest an increasing role of artificial intelligence in drug discovery and encourage the further clinical validation of the identified drugs. Our study contributes a valuable strategy that can be used for the rapid discovery of therapeutic solutions against an emerging health crisis.

This study presents a computational framework for identifying potential PLpro binders; however, the findings remain speculative in the absence of experimental validation. Future work will include in vitro and/or in vivo validation studies to experimentally assess the predicted interactions and binding affinities of the identified compounds.

## Figures and Tables

**Table 1:** The best docking scores (in kcal/mol) for predicting ligand binding sites. The ligands that were incorrectly predicted are highlighted in bold.

Ligand	Binding Site	Prediction	Average for S4	Average for SUb2
XR8	S4	S4	−8.740	−7.553
Y97	S4	S4	−8.560	−7.291
Y61	S4	S4	−8.80	−7.394
Y54	S4	S4	−8.647	−7.459
XT7	S4	S4	−9.080	−7.486
JW9	S4	S4	−7.433	−6.583
JWX	S4	S4	−8.173	−7.673
S88	S4	S4	−8.36	−8.104
GYX	S4	S4	−9.013	−8.176
XB5	S4	S4	−8.480	−7.018
XWO	S4	S4	−8.793	−6.893
XXW	S4	S4	−8.407	−6.950
XYI	S4	S4	−8.780	−7.161
XYR	S4	S4	−8.793	−7.037
Y2I	S4	S4	−8.687	−7.187
Y2N	S4	S4	−8.553	−6.728
Y2R	S4	S4	−8.393	−6.837
SR-01	S4	S4	−8.727	−8.114
TTT	S4	S4	−8.547	−7.415
Y41	S4	S4	−8.693	−7.631
Y94	S4	S4	−8.500	−7.550
Y95	S4	S4	−9.120	−7.877
Y96	S4	S4	−8.647	−7.303
VBY	S4	S4	−8.560	−7.4225
**9EI**	**S4**	**SUb2**	**−7.707**	**−7.857**
**L30**	**S4**	**SUb2**	**−7.440**	**−7.659**
A5I	SUb2	SUb2	−5.853	−5.997
A4O	SUb2	SUb2	−5.913	−6.088
T2	SUb2	SUb2	−5.987	−6.068
A7L	SUb2	SUb2	−5.987	−6.262
**A3X**	**SUb2**	**S4**	**−6.140**	**−5.890**
YRL	SUb2	SUb2	−4.967	−5.548
HBA	SUb2	SUb2	−4.720	−5.233

**Table 2:** Statistically important docking scores and their rank.

Docking Score From			Rank	Importance Contribution (%)
Docking Site	Cluster	Top Pose		
S4	2	4	1	7.45
S4	2	3	2	7.15
S4	2	2	3	6.92
S4	2	5	4	6.65
S4	2	1	5	5.01
S4	1	2	6	4.92
SUb2	2	2	7	4.56
S4	1	1	8	4.49
SUb2	2	5	9	3.64
S4	3	2	10	3.63
SUb2	2	1	11	3.42

**Table 3:** Drugs predicted to strongly bind PLpro as promising candidates for drug repurposing.

Drug	ATC Code	DrugBank ID	Use
Darifenacin	G04BD10	DB00496	Treat overactive bladder
Penbutolol	C07AA23	DB01359	Treat hypertension
Zafirlukast	R03DC01	DB00549	Treat asthma
Cromoglicic acid	R03BC01	DB01003	Treat asthma and allergies
Ponatinib	L01XE24	DB08901	Treat leukemia

**Table 4:** The X-ray crystallographic ligands of SARS-CoV-2 PLpro used in this study.

S. No.	Ligand	Binding Pocket	PDB ID	Reference
1	GRL0617	S3 and S4	7JIR	[43]
2	XR8-24		7LBS	[61]
3	XR8-65		7LOS	[61]
4	XR8-69		7LLZ	[61]
5	XR8-83		7LLF	[61]
6	XT7		7LBR	[61]
7	PLP_Snyder441		7JN2	-
8	PLP_Snyder494		7KOJ	-
9	PLP_Snyder495		7JIT	[43]
10	PLP_Snyder496		7KOK	-
11	PLP_Snyder530		7JIW	[43]
12	PLP_Snyder608		7SGU	-
13	PLP_Snyder630		7SGW	-
14	Jun9-72-2		7SDR	-
15	Jun9-84-3		7SQE	-
16	3k		7TZJ	[75]
17	S43		7E35	[76]
18	Jun12682		8UOB	[77]
19	Jun11941		8UUF	[77]
20	Jun12303		8UUG	[77]
21	Jun12199		8UUH	[77]
22	Jun12162		8UUU	[77]
23	Jun12197		8UUV	[77]
24	Jun12145		8UUW	[77]
25	Jun12129		8UUY	[77]
26	SR-01		8JUX	-
27	A5I	SUb2	7QCH	[55]
28	T2		7QCI	[55]
29	A7L		7QCK	[55]
30	A4O		7QCJ	[55]
31	A3X		7QCM	[55]
32	YRL		7OFS	[78]
33	HBA		7OFT	[78]

**Table 5:** Docking box dimensions.

Structure in MD Simulation	Representative Structure	Box Dimension (Å)
PDB ID 7LBR	R1	19.875 × 23.625 × 24.375
	R2	22.125 × 23.625 × 22.875
	R3	25.875 × 26.625 × 21.375
PDB ID 7QCI	R1	18.375 × 22.125 × 22.125
	R2	18.375 × 19.875 × 23.625
	R3	20.625 × 22.875 × 22.125

## Data Availability

All data generated or used in this study are provided at the journal’s website as Supplementary Materials.

## Supplementary Materials

The following supporting information can be downloaded at https://www.mdpi.com/article/10.3390/molecules30142985/s1, Supplementary Tables S1–S4, the Supplementary Data, and the Supplementary Information can be downloaded at the journal’s website. References [89,90,91] are cited in the Supplementary Materials.
