# Supplementary material for: Integrating Molecular Dynamics, Molecular Docking, and Machine Learning for Predicting SARS-CoV-2 Papain-like Protease Binders
Source: Molecules. 2025 Jul 16;30(14):2985. doi: 10.3390/molecules30142985 (PMC12300542; doi:10.3390/molecules30142985)
Supplement: Supplementary file 1 [file molecules-30-02985-s001.zip › Supplementary files/Supplementary Information.pdf]

Supplementary Documents

Integrating Molecular Dynamics, Molecular Docking and Machine Learning for Predicting SARS-CoV-2 Papain-like Protease Binders

Ann Varghese†, Jie Liu†, Tucker A Patterson, Huixiao Hong*

National Center for Toxicological Research, U.S. Food and Drug Administration, Jefferson, AR, 72079, USA

* Correspondence: huixiao.hong@fda.hhs.gov

† These authors made equal contributions.

Molecular Dynamics

1. **Two-step minimization**

Minimisation 1

```
initial minimisation solvent
 &cntrl
       imin   = 1,
       maxcyc = 10000,
       ncyc   = 5000,
       ntb    = 1,
       ntr    = 1,
       cut    = 10,
       restraintmask = ':1-318',!
       restraint_wt = 500
 /
```

Minimisation 2

```
initial minimisation solvent
 &cntrl
       imin   = 1,
       maxcyc = 10000,
       ncyc   = 5000,
       ntb    = 1,
       ntr    = 0,
       cut    = 10
 /
```

2. **Heating**

```
     Relaxation 1
 &cntrl
   imin=0, ntx=1, !
      ntb=1,cut=10.0, !
      ntp=0, !
      ntc=2, ntf=2 !
      ntt=3, gamma_ln=1.0, !
      tempi=0.0, !
      temp0=300.0,
      nstlim=100000, dt=0.001,!
      iwrap=1, !
      ioutfm=1, !
      ntpr=5000, ntwx=5000, !
      ntwr=50000, !
      ntr=1, restraint_wt=50.0, !
      restraintmask=':1-318',
      ig=-1, !
      nmropt=1, !
      /
```

```
&wt type='TEMP0', istep1=0, istep2=50000,value1=0.0, value2=300.0, /
&wt type='TEMP0', istep1=50000, istep2=100000,value1=300.0, value2=300.0, /
&wt type='END'  /
```

3. **Density equilibration**

```
Relaxation 2
&cntrl
  imin=0, ntx=5, irest=1, !
  ntb=2,cut=10.0, !
  ntp=1, pres0=1.0, !
  ntc=2,
  ntf=2,
  ntt=3, temp0=300.0, gamma_ln=1.0,
  nstlim=500000, dt=0.002,
  iwrap=1, ioutfm=1,
  ntpr=5000, ntwr=50000, ntwx=5000,
  ntr=1, restraint_wt=5.0, restraintmask=':1-318', !
  ig=-1,
  nmropt=1,
/
&wt type='TEMP0', istep1=0, istep2=500000,value1=300.0, value2=300.0 /
&wt type='END'  /
```

4. **Initial equilibration**

```
protein: equilibration
 &cntrl
     imin   = 0,
     irest  = 1,
     ntx    = 5,
     ntb    = 2,
     cut    = 10,
     ntp    = 1,
     ntc    = 2,
     ntf    = 2,
     taup    = 2.0,
     temp0  = 300.0,
     ntt    = 3,
     gamma_ln = 2.0,
     nstlim = 1500000, dt = 0.002,ioutfm=1,
     ntpr = 5000, ntwx = 5000, ntxo = 2
```

5. **Production simulation**

```
Production simulation
 &cntrl
  imin = 0, irest = 1, ntx = 7,
  ntb = 2, pres0 = 1.0, ntp = 1,
  taup = 2.0,
```

```
 cut = 10, ntr = 0,
 ntc = 2, ntf = 2,
 temp0 = 300.0,
 ntt = 3, gamma_ln = 2.0,
 nstlim = 500000000, dt = 0.002,ioutfm=1,
 ntpr = 10000, ntwx = 50000, ntwr = 10000, ntxo = 2, ntwv=-1
/
```

Molecular Docking (Sample input configuration file)

receptor = rigid.pdbqt
ligand = *_min.pdbqt

center_x = 34.150
center_y = 70.120
center_z = 40.690

size_x = 19.875
size_y = 23.625
size_z = 24.375

num_modes = 5
energy_range = 3
exhaustiveness = 32

Machine learning

```
################################################################################
# Model development
################################################################################
# LOOCV
################################################################################
import pandas as pd
import numpy as np
from itertools import product
from sklearn.ensemble import RandomForestClassifier
from sklearn.model_selection import LeaveOneOut, KFold, GridSearchCV
from sklearn.metrics import accuracy_score, classification_report
import time

# === Load Data ===
# Replace this with your actual Excel path
HOME = 'C:/Users/Jie.Liu1/OneDrive - FDA/Desktop/Desktop/project/ann-covid/'
df = pd.read_excel(HOME + 'data/Training-set.xlsx')

# Features and labels
X = df.descriptors.to_numpy()
y = df.label.to_numpy()

# === Define Parameter Grid ===
n_estimators = [50, 100, 200]
min_samples_split_vals = [2, 4, 6, 8, 10, 12]

param_grid = []
for n_est, split in product(n_estimators, min_samples_split_vals):
    leaf = split // 2
    if split >= 2 * leaf and leaf >= 1:
        param_grid.append({
            'n_estimators': [n_est],
            'min_samples_split': [split],
            'min_samples_leaf': [leaf]
        })

# === LOOCV ===
cv_outer = LeaveOneOut()
cv_inner = KFold(n_splits=5, shuffle=True, random_state=20)

predictions = []
y_true = []
probability = []
important_features = []
best_params_list = []
```

```
start_time = time.time()

for fold_idx, (train_idx, test_idx) in enumerate(cv_outer.split(X)):
    X_train, X_test = X[train_idx], X[test_idx]
    y_train, y_test = y[train_idx], y[test_idx]

    # Grid search on training data
    model = RandomForestClassifier(random_state=20)
    grid = GridSearchCV(model, param_grid, cv=cv_inner, scoring='accuracy', n_jobs=-1)
    grid.fit(X_train, y_train)

    best_model = grid.best_estimator_
    best_params_list.append(grid.best_params_)

    # Predict
    y_pred = best_model.predict(X_test)[0]
    y_true.append(y_test[0])
    predictions.append(y_pred)

    # Probabilities
    prob = best_model.predict_proba(X_test)[0]
    probability.append(prob)

    # Feature importance
    important_features.append(best_model.feature_importances_)

    print(f"Fold {fold_idx+1}/{len(X)} complete")

print(f"\nTotal LOOCV time: {time.time() - start_time:.2f} seconds")

# === Evaluate Performance ===
acc = accuracy_score(y_true, predictions)
print(f"\nLOOCV Accuracy: {acc:.4f}")
print("\nClassification Report:\n", classification_report(y_true, predictions))


################################################################################
# Random Forest Prediction on FDA-approved drug set
#################################################################################
import time
import pandas as pd
from sklearn.ensemble import RandomForestClassifier

DF1 = training_set
DF2 = FDA-approved drug set

start_time = time.time()
```

```
# Initialize outputs
predicted = []
probability = []
important_features = []

# === Prepare Training Data ===
train = DF1
X_train = train.iloc[:, 3:]
Y_train = train.iloc[:, 2]

# === Prepare Test Data ===
test = DF2.sort_values(by=['chem_id'], ascending=True)
X_test = test.iloc[:, 2:]

# === Train Random Forest ===
clf = RandomForestClassifier( n_estimators=200, min_samples_split=10,  min_samples_leaf=5,
   random_state=20)
clf.fit(X_train, Y_train)

# === Predict on Test Set ===
pred = clf.predict(X_test)
predicted.extend(pred)

# === Predict Probabilities ===
prob = clf.predict_proba(X_test)
probability.extend(prob)

# === Feature Importances ===
feature_important = clf.feature_importances_
important_features.append(feature_important)

            print("Total prediction time: %.2f seconds" % (time.time() - start_time))
```
